# Secondary contact between diverged host lineages entails ecological speciation in a European hantavirus

**DOI:** 10.1371/journal.pbio.3000142

**Published:** 2019-02-20

**Authors:** Moritz Saxenhofer, Sabrina Schmidt, Rainer G. Ulrich, Gerald Heckel

**Affiliations:** 1 Institute of Ecology and Evolution, University of Bern, Switzerland; 2 Swiss Institute of Bioinformatics, Quartier Sorge, Bâtiment Génopode, Lausanne, Switzerland; 3 Friedrich-Loeffler-Institut, Federal Research Institute for Animal Health, Institute of Novel and Emerging Infectious Diseases, Greifswald-Insel Riems, Germany; 4 German Center for Infection Research (DZIF), partner site Hamburg-Luebeck-Borstel-Insel Riems, Germany; Division of Clinical Research, UNITED STATES

## Abstract

The diversity of viruses probably exceeds biodiversity of eukaryotes, but little is known about the origin and emergence of novel virus species. Experimentation and disease outbreak investigations have allowed the characterization of rapid molecular virus adaptation. However, the processes leading to the establishment of functionally distinct virus taxa in nature remain obscure. Here, we demonstrate that incipient speciation in a natural host species has generated distinct ecological niches leading to adaptive isolation in an RNA virus. We found a very strong association between the distributions of two major phylogenetic clades in Tula orthohantavirus (TULV) and the rodent host lineages in a natural hybrid zone of the European common vole (*Microtus arvalis*). The spatial transition between the virus clades in replicated geographic clines is at least eight times narrower than between the hybridizing host lineages. This suggests a strong barrier for effective virus transmission despite frequent dispersal and gene flow among local host populations, and translates to a complete turnover of the adaptive background of TULV within a few hundred meters in the open, unobstructed landscape. Genetic differences between TULV clades are homogenously distributed in the genomes and mostly synonymous (93.1%), except for a cluster of nonsynonymous changes in the 5′ region of the viral envelope glycoprotein gene, potentially involved in host-driven isolation. Evolutionary relationships between TULV clades indicate an emergence of these viruses through rapid differential adaptation to the previously diverged host lineages that resulted in levels of ecological isolation exceeding the progress of speciation in their vertebrate hosts.

## Abstract 2

Like host, like virus; analysis of a natural hybrid zone in the European common vole reveals speciation processes in Tula hantaviruses triggered by evolutionary divergence in the rodent host lineages.

## Author summary

Natural biodiversity is driven by stochastic processes and evolutionary adaptation to ecological niches. In viruses, adaptation to specific hosts may cause diversification and eventually lead to the emergence of novel viruses. Here, we studied diversity in Tula orthohantavirus (TULV) in relation to evolutionary divergence in its natural rodent host, the European common vole (*Microtus arvalis*). In a geographical region in which two distinct evolutionary lineages in the common vole interact and interbreed (a hybrid zone), we found two substantially different TULV clades. Phylogenetic analyses revealed that the divergence among virus clades was likely triggered by a shift of an ancestral virus between the previously diverged host lineages in the hybrid zone. The strong association between virus clades and host lineages at a fine geographical scale results in effective separation of TULVs, despite incomplete reproductive isolation and frequent gene flow among local host populations. Virus genome sequences pointed to the amino-terminal part of the envelope protein as an important region for functional differentiation among these virus clades.

## Introduction

Evolutionary diversification has resulted in a myriad of virus species [1,2], but—despite their great importance as agents of diseases of humans, crops, and livestock—our understanding of their functional partitioning and distribution across hosts is strongly limited. The evolution of novel viral features and their molecular basis is best understood in laboratory and medical settings in which new virus types can arise quickly through genetic adaptation to specific host environments [3,4]. Ultimately, such adaptive processes may lead to ecological isolation of specialized virus types due to their reduced ability to use alternative hosts [5]. Heterogeneity in the environment of a virus population, e.g., through the simultaneous presence of different hosts, can also drive differential adaptation to divergent ecological niches resulting in “sympatric speciation” [6]. Although these principles of virus diversification have been demonstrated under controlled experimental conditions, our knowledge of the mechanisms that generate and maintain functionally distinct viruses in nature is very scarce [7].

The emergence of natural virus species is often investigated with a top-down approach reconstructing macroevolutionary patterns of diversification in the past [8]. Phylogenetic relationships of virus and host species can provide valuable information about long-term virus–host coevolution or ancient host-shift events [9]. However, divergent phylogenetic clades can be found within many natural virus species [10–15]. While genetic structuring below the species level could derive from selectively neutral processes like geographic isolation and random genetic drift, different virus clades may also represent functionally distinct entities resulting from differential adaptation in a heterogeneous host environment [16,17]. Still, the ecological conditions that promote virus divergence and potentially lead to the emergence of new virus species in nature are generally unclear.

Here, we implemented a bottom-up approach to investigate the origin of high genetic diversity in Tula orthohantavirus (TULV) and the role of host divergence as a potential driver of virus speciation. TULV (family Hantaviridae, formerly Bunyaviridae) is a horizontally transmitted, single-stranded RNA virus with a three-segmented genome of about 12 kilobases [18]. Genetic diversity in TULV is partitioned into several phylogenetic clades with allopatric or parapatric distribution across Europe [12,19]. On a large scale, the combined geographic ranges of specific TULV clades appear similar to those of several morphologically cryptic evolutionary lineages in its reservoir host, the common vole (*M*. *arvalis*), for which speciation processes started during or after the last glaciation [20–22]. We used a natural hybrid zone between evolutionary lineages in the rodent host to test on a local spatial scale whether TULV diversity was neutral or adaptive and determined the likely targets of host-induced isolation in the TULV genome. Implementing a methodical framework typically used to investigate speciation in sexual organisms [23], our comprehensive analyses demonstrate speciation processes in a hantavirus most likely initiated by a host shift between prediverged lineages of a single natural host species.

## Results

**Fig 1:**
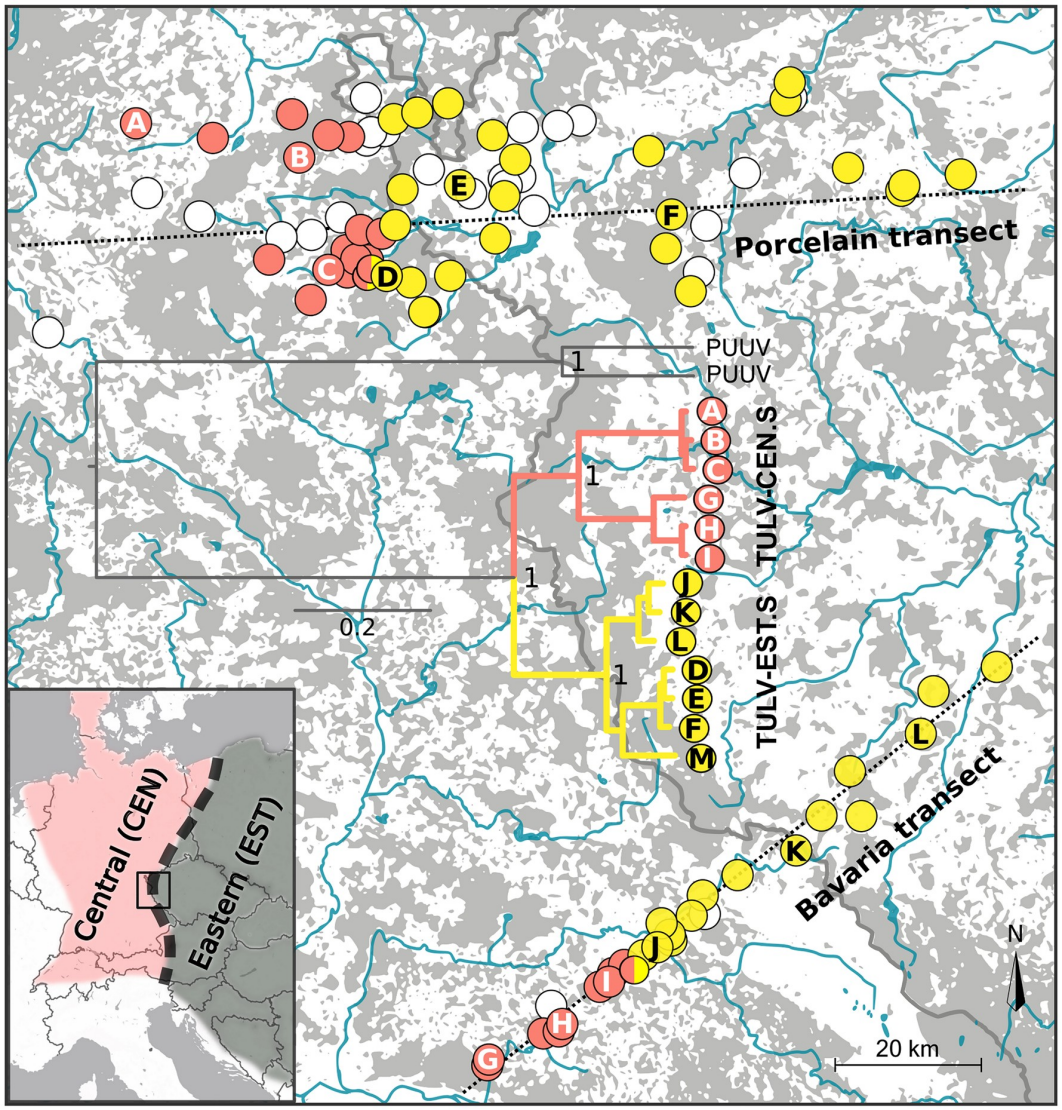
Distribution of two highly diverged TULV clades in a hybrid zone of their rodent reservoir host, the common vole *M*. *arvalis*. TULV-CEN.S (red) and TULV-EST.S (yellow) virus clades only co-occurred in two localities (bicolored) in the Porcelain transect and one in the Bavaria transect. The insert shows the position of the study region in Central Europe and the approximate distribution of the Central and Eastern evolutionary lineages in the common vole. Phylogenetic relationships based on complete TULV genome sequences are shown for samples from 12 localities across the host hybrid zone (A–L). The only published genome sequence from Eastern Czech Republic (M) was included for reference (see map in Fig 2). PUUV was used as outgroup (see S7 Table for accession numbers), and posterior probabilities of major nodes are indicated. Host sampling localities in which no TULV infections were detected are shown as white circles. Gray areas on the map represent forest (unsuitable habitat for common voles), and major water bodies are shown in blue. The border between Germany and the Czech Republic is indicated with a gray line. CEN, Central host lineage; EST, Eastern host lineage; PUUV, Puumala orthohantavirus; TULV, Tula orthohantavirus; TULV-CEN.S, Central South TULV; TULV-EST.S, Eastern South TULV.

**Fig 2:**
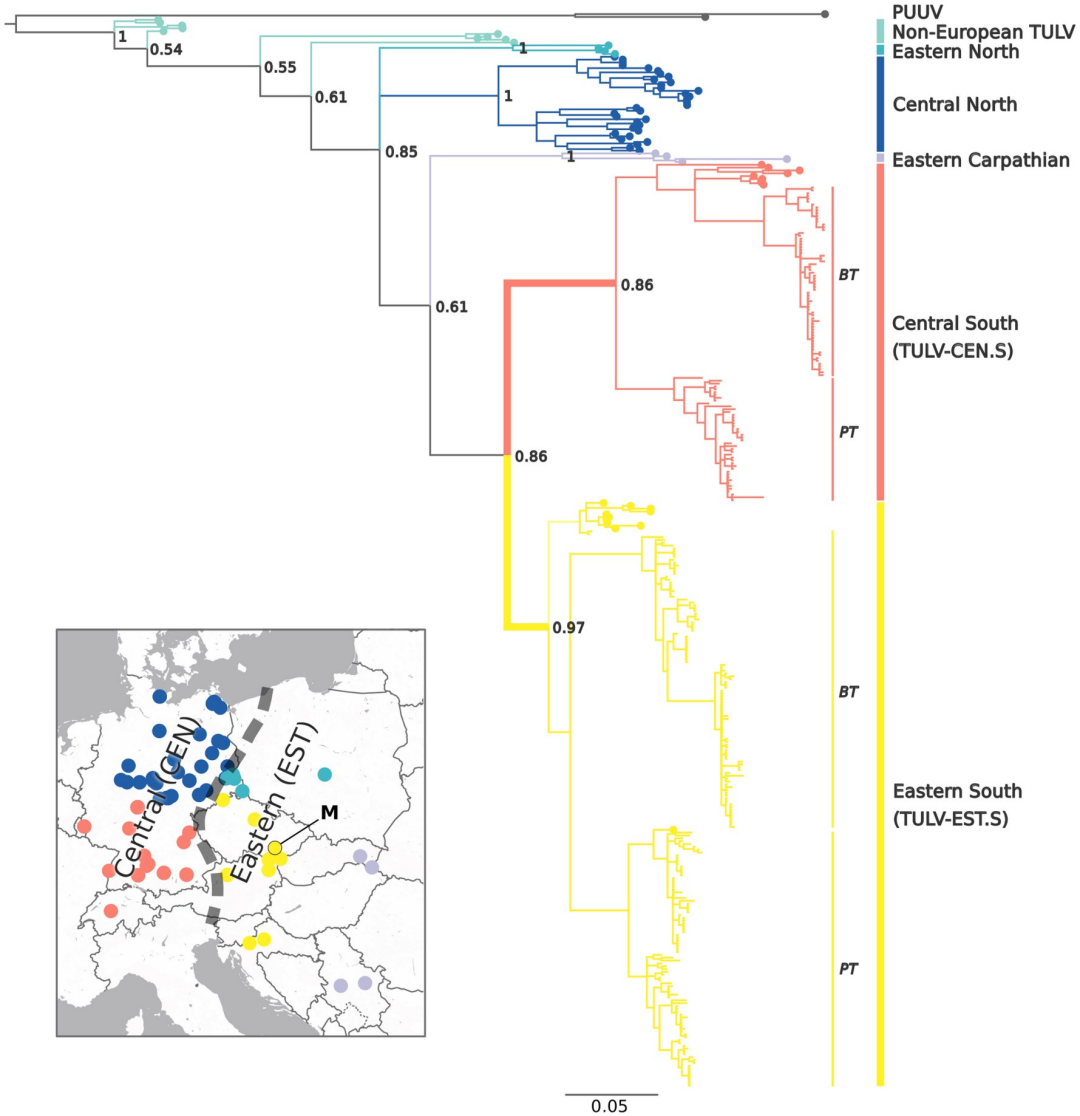
Evolutionary relationships and geographic distribution of major European TULV clades and associations to common vole host lineages. Phylogenetic analyses of novel TULV S-segment sequences (blunt tips) from the PT and the BT (see Fig 1) together with published reference sequences (round tips; 12) revealed two major clades in the common vole hybrid zone. Labels of TULV clades include the name of the respective host evolutionary lineage (CEN or EST; 19–21) and the geographic distribution of TULV clades within the host lineage (see insert and Fig 1). The insert shows the origin of TULV reference sequences across Europe (12; "M" marks the reference genome) with colors corresponding to major clades in the phylogenetic tree and the contact between CEN and EST host lineages as a dashed line. The close relationship among the TULV-CEN.S and TULV-EST.S clades (highlighted by a bold branch) together with the presence of additional, more diverged TULV clades in the same host lineages suggests secondary transmission between CEN and EST. Bayesian posterior probabilities are indicated for major nodes, and PUUV was used as outgroup. See S7 Table for reference and outgroup sequence accession numbers. BT, Bavaria transect; CEN, Central host lineage; EST, Eastern host lineage; PT, Porcelain transect; PUUV, Puumala orthohantavirus; TULV, Tula orthohantavirus; TULV-CEN.S, Central South TULV; TULV-EST.S, Eastern South TULV.

We found a very narrow geographic contact between two major phylogenetic clades of TULV in the hybrid zone between the Central and Eastern evolutionary lineages in the common vole (Fig 1). Molecular screening of 1,309 common voles from 109 locations detected 306 TULV-infected individuals at 65 locations in two replicate sampling transects. Viral sequences of the small genome segment (S-segment; see Methods) showed up to 37.6% sequence divergence at this local geographic scale. Phylogenetic analyses with reference sequences spanning the known TULV distribution assigned them to the two most recently diverged virus clades (Fig 2). We termed these TULV clades Central South (TULV-CEN.S) and Eastern South (TULV-EST.S) given their spatial association with different common vole host lineages and their respective geographic distribution relative to other, more diverged TULV clades in the northern range of the same host lineages (Fig 2). Sequences from the western parts of both transects (*n* = 111) were all most closely related with TULV strains found within the geographic distribution of the Central host lineage, whereas sequences from the eastern parts (*n* = 195) were all most similar to strains associated with the Eastern host lineage ([12]; Figs 1 and 2). Despite dense geographic sampling, we detected only at two adjacent locations in the Porcelain transect and one in the Bavaria transect TULV sequences from both viral clades (Fig 1 and S1 Fig), and two host individuals from these locations carried genome segments from different TULV clades.

**Fig 3:**
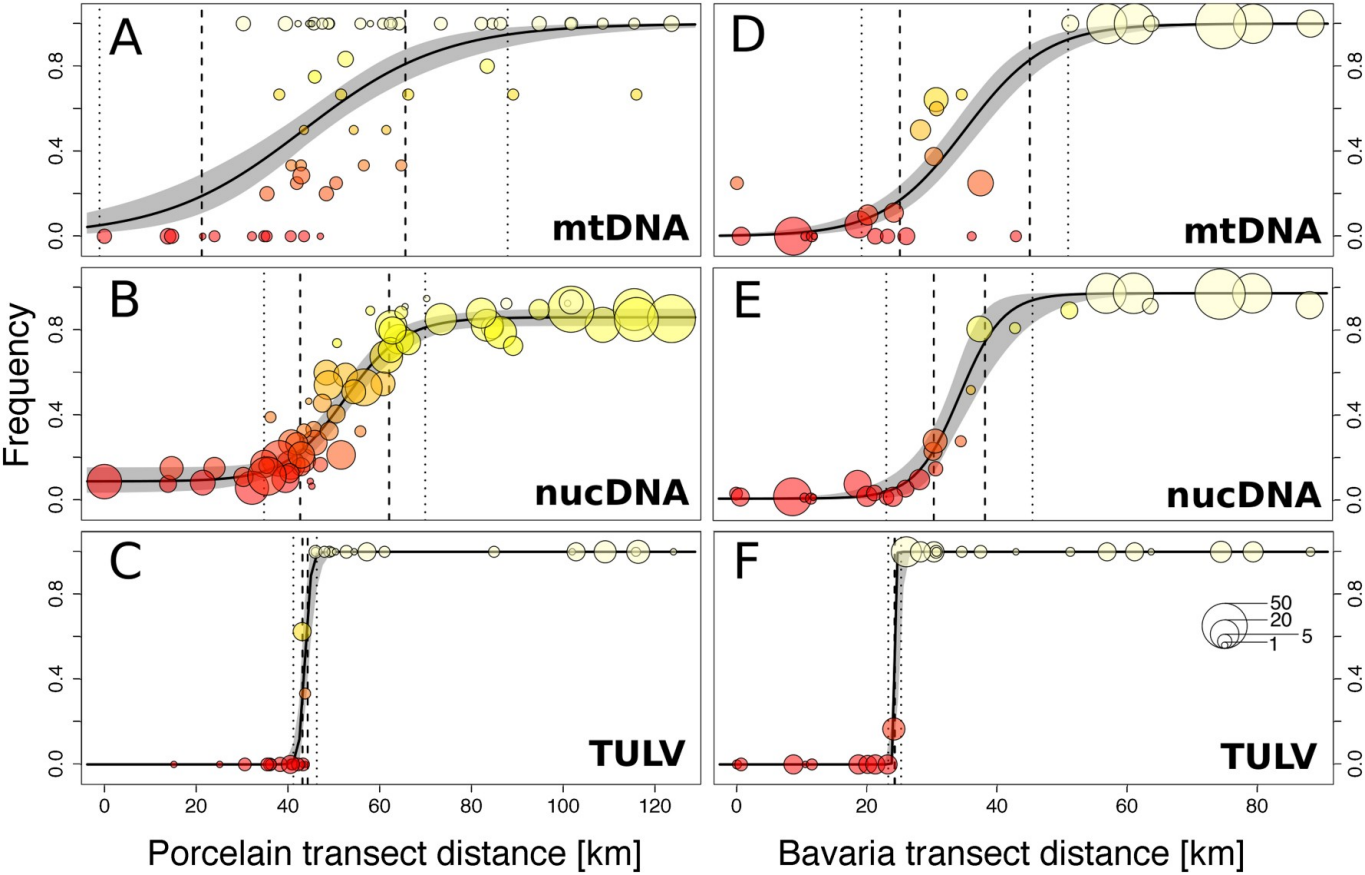
Geographic clines representing the transition between evolutionary lineages in the rodent host and phylogenetic clades in TULV along the Porcelain (A, B, and C) and Bavaria (D, E, and F) transects. The contact between the Central and the Eastern common vole lineages was characterized based on mtDNA (A and D) and nucDNA (B and E) markers, whereas for TULV clades partial S-segment sequences were phylogenetically assigned to TULV-CEN.S and TULV-EST.S clades (C and F; see Fig 2). The frequency per locality of Eastern host lineage (A, B, D, and E) and TULV-EST.S virus clade (C and F) is displayed starting from the western-most locality. Symbol sizes correspond to the number of samples and symbol colors to the genotype frequency per location. 95% credible cline regions are shown in gray. Cline widths are shown around the estimated cline center, with dotted and dashed lines indicating upper and lower width estimates (two log-likelihood support limits), respectively. mtDNA, mitochondrial DNA; nucDNA, nuclear DNA; TULV, Tula orthohantavirus; TULV-CEN.S, Central South TULV; TULV-EST.S, Eastern South TULV.

The spatial distribution of TULV clades in the common vole hybrid zone was strongly associated with the transition between the Central and Eastern evolutionary host lineages along both transects. Sequencing of the mitochondrial cytochrome *b* gene (mitochondrial DNA [mtDNA]; see Methods) and phylogenetic analyses identified 207 voles with Central lineage mtDNA in the western parts of the transects and 357 individuals with Eastern lineage mtDNA in the eastern parts (S2 Fig, Fig 3A and 3D). Bayesian clustering analyses of autosomal nuclear DNA (nucDNA; 16 microsatellite loci) of the host (*n* = 1,521) showed a clinal transition between two major genetic clusters in each transect with extensive admixture in individuals from locations in the core contact region (S3 Fig). Geographic clines demonstrated that the transition between TULV clades was spatially congruent but significantly steeper compared to the transition between evolutionary lineages in their hosts (Fig 3, Table 1). The widths of the clines in TULV were estimated as 0.3 km (two log-likelihood support limits: 0.0–2.0 km) in the Bavaria transect and 2.5 km (1.1–5.1 km) in the Porcelain transect (Table 1). Depending on transect and marker type, the widths of the zones of hybridization and admixture between host lineages were estimated between 13.2 km (8.6–19.3 km) and 61.1 km (44.4–89.0 km), which is eight to 40 times wider than the virus contact (Fig 3, Table 1). Sequences from parts of the medium (M; *n* = 54) and large (L; *n* = 60) genome segments of TULV sampled in and around the locations where the virus clades were in contact (Fig 1) demonstrated that the divergence between TULV clades in all segments was highly spatially consistent (S4 and S5 Figs).

**Table 1:** Geographic transition between host lineages and TULV clades.

	Width			*p*-Values		
	mtDNA	nucDNA	TULV	mtDNA–nucDNA	mtDNA–TULV	nucDNA–TULV
**PT**	61.1 (44.4–89.0)	25.9 (19.4–35.1)	2.5 (1.1–5.1)	0.010	0.001	0.002
**BT**	25.2 (20.0–31.7)	13.2 (8.6–19.3)	0.3 (0.0–2.0)	0.007	0.002	0.003

Width of the mixture zone between host lineages based on the transition of mtDNA and nucDNA or TULV clades along the PT and BT (see text). Width estimates are given in kilometers with two log-likelihood support limits in brackets. *p*-Values refer to likelihood ratio tests of differences in width between estimates from the same transect (see text).

**Abbreviations**: BT, Bavaria transect; mtDNA, mitochondrial DNA; nucDNA, nuclear DNA; PT, Porcelain transect; TULV, Tula orthohantavirus.

**Fig 4:**
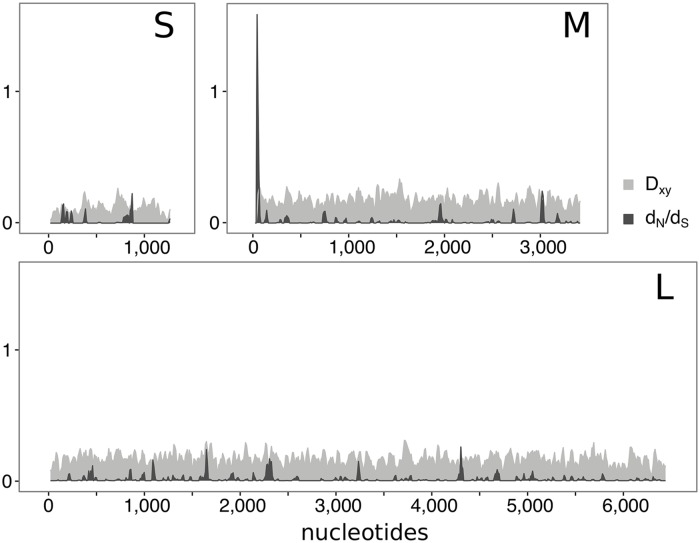
Sliding-window analyses in the coding regions of the small, medium, and large TULV genome segment. For 12 TULV genomes (six from the TULV-CEN.S and six from the TULV-EST.S clade; see Fig 1), the ratio of nonsynonymous to synonymous substitutions and the average number of nucleotide substitutions per site were calculated for sequence windows of 30 nucleotides shifted by 10 nucleotides. x-Axes indicate nucleotide positions along the coding sequence of each genome segment. d_N_/d_S_, ratio of nonsynonymous to synonymous substitutions; D_xy_, average number of nucleotide substitutions per site; L, large genome segment; M, medium genome segment; S, small genome segment; TULV, Tula orthohantavirus; TULV-CEN.S, Central South TULV; TULV-EST.S, Eastern South TULV.

High-throughput sequencing of six TULV-CEN.S and six TULV-EST.S genomes (three from either side in both transects, see Fig 1; S1 Table) confirmed strong genome-wide divergence between the clades, with an overall nucleotide diversity of 0.144 in coding regions (Fig 4). The only complete TULV genome publicly available to date (see Methods) clustered in phylogenetic analyses consistently with the TULV-EST.S genomes from both transects despite its origin about 250 km to the east (Figs 1 and 2). Our analyses provided no evidence for recombination between different TULV clades. However, recombination breakpoints located between the M and L TULV genome segments were detected within the TULV-EST.S clade (Porcelain transect; *p* = 0.0074; Bavaria transect; *p* = 4 × 10^−5^; S6 Fig). TULV-CEN.S and TULV-EST.S genomes from the contact zone showed fixed differences at 579 nucleotide positions in coding regions (11,169 nucleotides total) of which 539 (93.1%) were synonymous and only 40 (6.9%) altered the amino acid sequence of the encoded protein (Table 2 and S2 Table). This very low ratio of nonsynonymous to synonymous substitutions is consistent with pervasive purifying selection acting overall on these viral genomes. However, statistical tests for selection revealed signals of diversifying selection between TULV clades in specific regions of the M- and L-segments (S3 Table). Branch-site models and mixed-effect models of evolution in the softwares PAML and HYPHY (see Methods) identified individual codons under potential positive selection, but only estimates for codon 17 in the M-segment were significant with both methods (S4 and S5 Tables). A sliding-window analysis showed an accumulation of nonsynonymous but not of synonymous differences in the same region of the M-segment encoding the viral envelope glycoprotein, which resulted in a conspicuously high local d_N_/d_S_ ratio (Fig 4). Taken together, the signal of diversifying selection on a few TULV amino acid positions suggests that these sites could be responsible for effective ecological isolation of differentially adapted virus clades.

**Table 2:** Nucleotide polymorphisms in 12 TULV genomes from the common vole hybrid zone between the Central and Eastern evolutionary lineage.

		TULV-CEN.S (*n* = 6)			TULV-EST.S (*n* = 6)			Between clades (*n* = 12)		
	Length CDS	P_n_	P_s_	d_N_/d_S_	P_n_	P_s_	d_N_/d_S_	D_n_	D_s_	d_N_/d_S_
**S-segment**	1,287 nts	2	176	0.003	3	156	0.006	6	42	0.007
**M-segment**	3,423 nts	33	728	0.008	9	408	0.006	5	227	0.004
**L-segment**	6,459 nts	58	1,193	0.008	24	1,076	0.006	29	270	0.007
**Concatenated segments**	11,169 nts	93	2,097	0.009	36	1,640	0.006	40	539	0.007

Gene CDSs of the small, medium, and large TULV genome segments were analyzed separately and as concatenated sequences. The number of polymorphic nonsynonymous and synonymous positions within the TULV-CEN.S and TULV-EST.S virus clades are indicated. Nucleotide divergence between TULV clades is quantified as nonsynonymous and synonymous positions with variants private to one viral clade. The ratio of nonsynonymous to synonymous substitutions is given for individual clades and comparing all genomes.

**Abbreviations**: CDS, coding sequence; D_n_, number of nonsynonymous positions with variants private to one viral clade; D_S_, number of synonymous positions with variants private to one viral clade; d_N_/d_S_, ratio of nonsynonymous to synonymous substitutions; L-segment, large genome segment; M-segment, medium genome segment; nts, nucleotides; P_n_, number of polymorphic nonsynonymous positions; P_S_, number of polymorphic synonymous positions; S-segment, small genome segment; TULV, Tula orthohantavirus; TULV-CEN.S, Central South TULV; TULV-EST.S, Eastern South TULV.

## Discussion

Our local-scale analyses demonstrate a stage in the speciation process of a natural hantavirus in which ecological separation in the virus precedes complete reproductive isolation in the host (Fig 3). The patterns of evolutionary divergence between TULV clades revealed by the complementary top-down perspective (Fig 2) indicate that functional divergence among TULV-CEN.S and TULV-EST.S was established after a host shift in the secondary contact between previously diverged host lineages.

Allopatric divergence of common vole lineages [20–22] has likely provided the evolutionary substrate for adaptive separation and the emergence of ecologically isolated TULV clades. At first sight, this may appear like the result of codivergence between virus clades and host lineages—a pattern once assumed to be typical for the evolution of hantaviruses in general [24–26]. However, despite the respective association of TULV-CEN.S and TULV-EST.S with the Central and Eastern common vole lineages, additional more divergent TULV clades are present in the northern parts of the ranges of the same host lineages (Fig 2). This is in conflict with a scenario of allopatric virus–host codivergence (e.g., in glacial refugia) under which virus clades in the same host lineage should be closest related [27]. It is therefore more likely that only the postglacial formation of the vole hybrid zone [21,22] provided the opportunity for a transmission of the TULV-CEN.S and TULV-EST.S ancestor between host lineages with subsequent specialization. Error prone replication and very large effective population sizes in RNA viruses generally facilitate rapid diversification and adaptation to novel hosts [28]. In heterogeneous host environments, differentially specialized viruses may emerge [6,29–31] if the improved adaptation to one ecological resource outweighs the reduced ability to use an alternative resource, representing an evolutionary tradeoff [32]. The secondary contact between two prediverged common vole lineages after postglacial recolonization [20–22] strongly increased local heterogeneity in the TULV host environment. TULV-CEN.S and TULV-EST.S have therefore likely diverged as a direct consequence of diversifying natural selection in the secondary contact zone.

The congruence of spatial separation (Fig 1) and sequence divergence across all genome segments (Figs 2 and 4, S4 and S5 Figs) indicates pervasive barriers between TULV-CEN.S and TULV-EST.S despite frequent current gene flow through hybridization among host lineages (S3 Fig). Recombination or genome reassortment has been suggested for TULV and other hantaviruses [33–36]. We found limited evidence for reassortment of TULV genome segments between TULV-EST.S sequences but not in TULV-CEN.S or between clades (S6 Fig). While recombination or genome reassortment may play a role in TULV evolution in general, our data from two replicate transects demonstrate that their impact on the integrity of TULV-CEN.S and TULV-EST.S clades in this virus contact zone is insignificant. Both clades co-occurred in three host populations (Figs 1 and 3 and S1 Fig), and two voles from these populations showed evidence for co-infection and thus opportunity for potential virus reassortment. However, clade affiliation was consistent for all TULV segments from all other individuals (among these 23 from mixed TULV populations) including 12 complete genomes (Fig 1). It remains to be shown whether reassortment is generally impeded between differentially adapted viruses in co-infected hosts, e.g., by genetic incompatibilities [37]. Overall, the strong and pervasive evolutionary separation between TULV-CEN.S and TULV-EST.S is, in several aspects, consistent with the criteria for complete biological speciation [38,39], despite incomplete reproductive isolation among the host lineages [21].

The comparative analysis of admixture patterns at an ecologically relevant spatial scale also provides clues on the adaptive landscape [e.g., 40,41] of the TULV clades. Historical and ongoing gene flow in the common vole hybrid zone has resulted in a gradual and relatively smooth transition between two divergent TULV host environments (Fig 3A, 3B, 3D and 3E). We see no possibility for extrinsic factors such as landscape connectivity or climatic differences at a geographic scale of a few kilometers to block TULV transmission without blocking dispersal in the host as well. The local frequencies of TULV-CEN.S and TULV-EST.S are therefore informative about their relative fitness in host populations. Very steep transitions between virus clades (Fig 3C and 3F, Table 1) indicate strongly isolated fitness optima such that small overall changes in host allele or genotype frequencies translate to a major shift in the TULV adaptive landscape. Ecological separation between TULV-CEN.S and TULV-EST.S is therefore probably mediated by a matching-allele type of virus–host interaction [42]. Vole populations of intermediate admixture were predominantly infected by the TULV-EST.S clade, shifting the virus contact towards the Central side of the host hybrid zone (Fig 3). This is consistent with unequal lineage-specific selective effects, e.g., through genetic dominance of Eastern host lineage alleles, mediating higher general fitness of TULV-EST.S in the heterozygous background of admixed hosts.

Adaptive divergence between TULV-CEN.S and TULV-EST.S is apparently driven by a low number of amino acid differences (Table 2 and S2 Table), probably affecting key functions of the virus–host interactions [5,6]. TULV is frequently detected as spillover infection in several arvicoline rodent species [12,43,44], which demonstrates its ability to infect hosts that are genetically much more diverged than common vole lineages. Adaptive divergence between TULV-CEN.S and TULV-EST.S is therefore mediated by a mechanism affecting virus persistence rather than initial host infection. Higher relative fitness of TULV-CEN.S in the Central host lineage and vice versa has probably resulted in host-induced ecological isolation. The patterns of genomic variation suggest a particular importance of the 5′-terminal coding region of the M-segment for differential host adaptation of TULV-CEN.S and TULV-EST.S (Fig 4, Table 2 and S2–S5 Tables). The M-segment encodes the viral glycoprotein precursor (GPC) and its cotranslational cleavage products, which are crucial for virus–host interactions [45]. The N-terminal part of the GPC represents a signal peptide in other hantaviruses [18], but the exact GPC processing in TULV has not been investigated. Adaptive variation in regulatory elements can mediate immune escape through interference with the intracellular processing of virus-derived proteins [46], e.g., via the major histocompatibility complex (MHC) class I antigen presentation pathway [47]. Alternatively, if the signal sequence is not fully cleaved [48], this region may directly interact with host receptors or components of the immune system as part of the N-terminal ectodomain in the mature viral glycoprotein spike complex [49].

## Conclusion

Here, we demonstrated that the emergence of two highly diverged clades in TULV was likely driven by adaptive differentiation rather than neutral processes mediated by relatively few genetic changes in the viral genome. Similar processes may act during the establishment of genetic structuring and potentially speciation in other viruses, including human pathogenic rabies virus [10], Lassa arenavirus [11,14], or Puumala orthohantavirus (PUUV) [13], but comparable data from ecologically relevant spatial scales are scarce. Taking potential population structure and movements of hosts in natural systems explicitly into account requires considerable effort [50] but offers the potential to bridge the gap to experimental insights on virus evolution and lead to a more complete understanding of the emergence of novel virus species.

## Methods

### Ethics statement

Rodent trapping occurred after ethical evaluation and approval by the Bernese cantonal commission on animal experimentation under permits BE-90/10 and BE-33/14 issued by the cantonal veterinary offices.

### Samples

Common voles were sampled in the border region between Germany and the Czech Republic, where earlier analyses detected a relatively narrow hybrid zone between the evolutionary lineages Central and Eastern in the Bavaria transect [21]. The current study increased the density of sampling in the Bavaria transect considerably and added the completely new and more extensive transect Porcelain approximately 100 km to the north (Fig 1). Sampling occurred particularly at the center of the hybrid zone at ecologically relevant spatial scales such that distances between localities could be covered by individual voles within a few hours [51–53], and gene flow patterns suggest frequent dispersal (Fig 3). The Porcelain transect was 124 km long, covering 65 localities and 1,234 common vole individuals, and the Bavaria transect (88 km) contained 27 sampling localities from which 778 voles were obtained. Voles were trapped with snap traps and stored at −20 °C immediately after collection. DNA was extracted from a tissue sample following a standard phenol-chloroform protocol.

### Host genotyping

We used mtDNA and nucDNA markers to determine the spatial distribution of the evolutionary lineages in the vole hosts and assess the admixture status. For mtDNA, we sequenced a part of the cytochrome *b* gene following [54] for at least three individuals per population if possible, resulting in 253 novel sequences total. Additional mtDNA data for the Bavaria transect was taken from Beysard and Heckel [21]. Phylogenetic analysis was done using MrBayes version 3.2.6 [55] on the CIPRES platform [56]. Implementing reversible-jump sampling over the entire general time-reversible substitution model space [57], Metropolis-coupled Markov chain Monte Carlo (MCMC) sampling was performed for 10^7^ generations in four independent runs comprising four chains. After discarding a burn-in fraction of 25%, samples were recorded every 10^3^ generations. Host individuals were assigned to evolutionary lineages according to phylogenetic clustering of their mtDNA sequences with reference data as in [58] (S2 Fig). Analogous to Beysard and Heckel [21] and Beysard and colleagues [59], the structure of the hybrid zone and admixture patterns were inferred with nucDNA data from 16 microsatellite loci [20,60,61] using Bayesian genetic clustering with Structure version 2.3.4 [62]. We analyzed a total of 1,521 individuals—1,015 from the Porcelain transect and 506 from the Bavaria transect separately—implementing the admixture model with correlated allele frequencies [63] in four independent runs of an MCMC search (10^6^ generations after 10^5^ burn-in iterations) for each number of clusters (K = 1–10). The optimal number of clusters was subsequently determined using the method from Evanno and colleagues [64] implemented in Structure Harvester [65]. In agreement with earlier analyses in the Bavaria transect [21], this clearly supported two genetic clusters in both transects.

### TULV screening and strain assignment

Common voles of at least 20 g body weight were screened for TULV infection because younger individuals are typically protected by maternal antibodies [66]. TULV infection was assessed by amplification of 540 nucleotides of the gene encoding the viral nucleocapsid protein on the S-segment using the RT-PCR assay described in [67]. RNA extractions from lung tissue and sequencing of amplified fragments followed Schmidt and colleagues [12]. Phylogenetic analyses of TULV sequences were performed as described above, while evolutionary parameters of combined first + second and third codon positions were estimated independently. Sequences from two PUUV strains (S7 Table) served as outgroups, and complete coding sequences (CDSs) of all three genome segments were concatenated for the analysis of TULV genomes (Fig 1). TULV samples were assigned to larger phylogenetic clades with allopatric or parapatric distribution in Europe using sequences from Schmidt and colleagues [12] as references. For samples originating from locations in which TULV-CEN.S and TULV-EST.S clades occurred together and from adjacent populations (50 samples from 9 populations total), sequences were additionally generated from the M and L genome segments using primers C1 and C2 [43] and HanLF1 and HanLR1 [68] according to their respective protocols. Phylogenetic reconstruction was done as described above. We estimated the maximum genetic divergence between TULV strains sampled in the same population as the largest distance along branches (sum of branch lengths) connecting a sequence pair from the same location in a phylogenetic tree ([19]; S1 Fig).

### Cline analysis

Geographic clines were inferred along one-dimensional transect axes crossing the contact zones for mtDNA and nucDNA host markers and TULV S-segment data using the HZAR package [69]. Transect axes were defined as the projection line minimizing the sum of rectangular distances from individual sampling locations to that line. The distance between locations along the cline axis is the distance between projection points. Three cline models with increasing levels of parametrization were implemented: model 1 (free cline center and width; cline ends fixed to minimum and maximum observed frequency), model 2 (additional free minimum and maximum frequency), and model 3 (additional free parameters for independent exponential tails). Likelihood scores of the cline models were compared for each data set, and cline parameters were estimated for the model with highest likelihood performing 10^5^ generations of an MCMC search in three independent chains after 10^4^ burn-in iterations. Cline concordance and coincidence were inferred with a likelihood ratio test [70,71]. For mtDNA, nucDNA, and TULV data, the likelihood profile for the parameters’ cline center and width were explored. Model likelihoods were determined for 20 fixed values for cline center and width, respectively. Cline centers were fixed to equal steps between kilometers 20 to 80 and 20 to 60 along the transect axes and cline width to values between 1 to 60 and 1 to 40 km for the Porcelain and Bavaria transect, respectively. The test statistic was calculated as two times the difference in log-likelihood between a null model assuming concordance and the alternative model supporting different cline widths for different markers [71]. Significance was determined based on a χ^2^ distribution with one degree of freedom equal to the difference in parameters estimated between the alternative model and the null model.

### TULV genome sequencing and assembly

Twelve TULV positive samples were chosen for shotgun sequencing of the virus genome based on their origin relative to the TULV contact in each transect. Libraries were prepared from total RNA extracted from lung tissue with no pre-amplification of the viral genome using the Illumina TruSeq kit. Libraries were sequenced on 1.5 lanes of an Illumina MiSeq at 2 × 300 bp paired-end and one lane of a HiSeq3000 at 100 bp single-end (see S1 Table). In every sequencing run, libraries from both TULV clades were analyzed together, and sequence reads of the same sample from multiple lanes were combined. Reads were trimmed using default settings in Trimmomatic version 0.32 [72] and initially mapped to the only available full TULV genome (GenBank accession numbers NC005227 S-segment, NC005228 M-segment, NC005226 L-segment) using the mem algorithm of BWA version 0.7.15 [73] with parameters–B 3 –k 14. The MarkDuplicates function using default settings and AddOrReplaceGroups of Picard version 1.99 (http://broadinstitute.github.io/picard) were implemented to remove duplicates, and BAM files were subject to local realignment using GATK version 3.7.0 [74]. Genotypes were called with GATK’s UnifiedGenotyper and the following parameters:—output_mode EMIT_ALL_SITES—min_base_quality_score 10—standard_min_confidence_threshold_for_calling 10. FastaAlternateReferenceMaker from GATK was used to generate individual consensus sequences for every genome assembly. Once a first consensus sequence was obtained, raw sequencing reads from the individual samples were mapped back to their own consensus sequence using BWA’s mem with more stringent parameters–B 4 –k 19. For samples that yielded multiple contigs per genome segment in the first assembly, the consensus sequence of another sample from the same transect and of the same TULV clade was used to fill a few remaining gaps between contigs. A second consensus sequence was then generated as described above. Sanger sequences from the partial S-segment were used to confirm consensus sequences indicating a mismatch every 4.63 × 10^−4^ nucleotides on average. Mapping statistics were collected implementing samtools version 1.3.1 [75] stats command, and coverage was inferred with DepthOfCoverage from GATK. Details on assembly statistics and sequence coverage are given in S1 Table. We checked for a potential TULV clade-specific assembly bias by performing additional reference-guided assemblies for every sample using consensus sequences from both transect ends as references. Genome sequences from both assemblies were identical, providing no evidence for a reference-induced bias. Gene CDSs from all three genome segments were concatenated for further analyses. Multiple sequence alignments were performed in BioEdit [76] using CLUSTAL W [77]. Phylogenetic reconstruction was done in MrBayes as described above using PUUV as outgroup.

### Sequence diversity and selection inference

DnaSP version 5 [78] was used to estimate genome-wide nucleotide sequence diversity and the number of polymorphic sites and to perform sliding-window analyses implementing the Jukes and Cantor correction [79]. A full exploratory recombination scan was performed on concatenated gene CDSs of all three genome segments in RDP4 [80] implementing the RDP, GENECOV, and MaxChi methods. Recombination events detected in the initial scan were re-analyzed using all available methods, and *p-*values were taken from MaxChi. We used the codeml program from the PAML package version 4.8 [81] to test for signals of selection implementing the branch-site model [82] and clade model C [83]. Model fitting was based on tree topologies inferred using RAxML version 8.2.10 [84] on the CIPRES platform [56]. Model likelihoods were compared to a null hypothesis using a likelihood ratio test with a χ^2^ distribution. Comparisons between random sites models were used to test for positively selected codons (M2a versus M1a, and M8 versus M7 and M8a) (S6 Table). Sites under positive selection were identified by Bayes’ Empirical Bayes analysis implemented in PAML. To incorporate rate variation at synonymous sites, we also investigated positive selection using the FUBAR [85] and MEME [86] methods in HYPHY [87] on the Datamonkey webserver [88]. Sites with posterior probabilities > 0.8 or *p* < 0.1 were deemed to be positively selected.

## Supporting information

S1 Fig Maximum phylogenetic divergence (see Methods) among TULV partial S-segment sequences sampled within individual host populations. Genetic divergence was plotted against the projected geographic distance in kilometers along the axes of the Porcelain (A) and Bavaria (B) transects starting from the western-most locations. Symbol sizes are proportional to the number of TULV sequences obtained from a location. S-segment, small genome segment; TULV, Tula orthohantavirus.
(EPS)

S2 Fig Bayesian phylogenetic tree of mitochondrial cytochrome *b* gene sequences. Novel sequences (blunt tips) from the Porcelain and Bavaria transects were assigned to the Central (red) and Eastern (yellow) evolutionary lineages in the common vole using published reference sequences (round tips). Bank vole (*Myodes glareolus*) sequences were used as outgroup. Posterior node probabilities are indicated for major nodes.
(EPS)

S3 Fig Genetic structure in common voles (*M*. *arvalis*) in the Porcelain (A) and Bavaria (B) transects across the hybrid zone between the Central and Eastern evolutionary lineages based on autosomal microsatellite markers. Each bar represents the individual probability of membership as determined with the software STRUCTURE to the Central (red) or Eastern (yellow) common vole evolutionary lineages. Individuals are grouped by sampling location separated by vertical black lines. Numbers represent geographic distances in kilometers along the cline axes from the western-most location of each transect.
(EPS)

S4 Fig Phylogenetic relationships between TULV M-segment sequences (366 nucleotides). Novel sequences (*n* = 54) from PT and BT (blunt tips) cluster with published sequences (round tips, S7 Table) from the TULV-CEN.S (red) and TULV-EST.S (yellow) clades. PUUV was used as outgroup; posterior probabilities for major nodes are indicated. BT, Bavaria transect; M-segment, medium genome segment; PT, Porcelain transect; PUUV, Puumala orthohantavirus; TULV, Tula orthohantavirus; TULV-CEN.S, Central South TULV; TULV-EST.S, Eastern South TULV.
(EPS)

S5 Fig Phylogenetic tree based on 283 nucleotides of the TULV L genome segment. Published sequences (round tips, S7 Table) were used to assign 60 novel sequences (blunt tips) from PT and BT to the TULV-CEN.S (red) and TULV-EST.S (yellow) clusters. Note that no sequences from the TULV clade Eastern North are available for this genomic region. For major nodes, posterior probabilities are indicated; PUUV was used as outgroup. BT, Bavaria transect; L, large; PT, Porcelain transect; PUUV, Puumala orthohantavirus; TULV, Tula orthohantavirus; TULV-CEN.S, Central South TULV; TULV-EST.S, Eastern South TULV.
(EPS)

S6 Fig Pairwise sequence identity between potentially reassorted TULV-EST.S genome sequences from the Porcelain (A) and Bavaria (B) transects resulting from a sliding-window analysis with the software RDP4. Pairwise identity between J, K, and L (red: J–K; blue: J–L; orange: K–L) and D, E, and F (red: D–E; blue: D–F; orange: E–F) sequences is shown on the y-axis (see Fig 1; window size = 30 nucleotides). The x-axis indicates nucleotide positions along concatenated CDS of the TULV genome. Vertical gray lines mark boundaries between the S, M, and L genome segments, and the dashed line marks the reassortment break point inferred with RDP4. CDS, coding sequence; L, large genome segment; M, medium genome segment; S, small genome segment; TULV, Tula orthohantavirus; TULV-EST.S, Eastern South TULV.
(EPS)

S1 Table Overview of TULV genome assemblies. Sample: sample name corresponding to Fig 1; sequencing: Illumina read length and pairs specification; total nr of reads: number of reads after duplicates were removed (see Methods section); percentage of reads mapped: fraction of primary aligned reads to the reference sequence; nr of reads: number of reads mapped to the genome segment; nr of contigs: number of regions covered by overlapping sequence reads; average coverage: mean number of reads per nucleotide; median coverage: median number of reads per nucleotide; % ORF covered: fraction of open reading frame sequences; % nucleotide cov ≥ 15: fraction of the sequenced genome covered at minimum 15× depth; alternative reference: reference genome used in the final assembly for samples for which primary assemblies did not yield a full genome sequence. ORF, open reading frame; TULV, Tula orthohantavirus.
(XLSX)

S2 Table Amino acid positions in nucleocapsid protein (S-segment), GPC (M-segment), and RNA-dependent RNA polymerase (L-segment) with variants private to six TULV-CEN.S (A–C and G–I) or six TULV-EST.S (D–F and J–L) genomes (see Fig 1). The region of high amino acid diversity in the M-segment–encoded envelope glycoprotein is indicated with bold letters. Colors correspond to biochemical properties of amino acids: yellow–nonpolar, green–polar, light blue–basic, and dark blue–acidic. GPC, glycoprotein precursor; L-segment, large genome segment; M-segment, medium genome segment; S-segment, small genome segment; TULV, Tula orthohantavirus; TULV-CEN.S, Central South TULV; TULV-EST.S, Eastern South TULV.
(XLSX)

S3 Table Inference of divergent natural selection between TULV clades. Clade model C implemented in the software PAML was fitted to individual TULV genome segments with data partitioned according to TULV-CEN.S and TULV-EST.S phylogenetic clades. The ratios of nonsynonymous to synonymous substitutions (*ω* = d_N_/d_S_) are indicated for different site classes (*ω*_0_, *ω*_1_, and *ω*_d_) such that the divergent site class *ω*_d_ evolves differently among partitions. The proportion of each site class is given in parenthesis. lnL was compared to the null-model M2a_rel, which does not allow divergence of *ω* in the third site class. The number of model parameters and the ratio of transitions to transversions are indicated, as well as the *D* value of the LRT and *p*-values deduced from a χ^2^ distribution with 1 degree of freedom. CmC, clade model C; d_N_/d_S_, ratio of nonsynonymous to synonymous substitutions; L, large genome segment; lnL, model likelihood; LRT, likelihood ratio test; M, medium genome segment; np, the number of model parameters; S, small genome segment; TULV, Tula orthohantavirus; TULV-CEN.S, Central South TULV; TULV-EST.S, Eastern South TULV; κ, transition to transversion ratio.
(XLSX)

S4 Table Results from BrS analyses performed on individual TULV genome segments. The data were partitioned according to TULV-CEN.S and TULV-EST.S phylogenetic clades with the indicated partition analyzed as FG where the BG contained the remaining taxa. The nonsynonymous to synonymous substitutions ratios (*ω* = d_N_/d_S_) are indicated for different site classes and different partitions with the proportion of sites allocated to each site class stated. The branch-site models were compared to a null model (BrS_null) in which *ω*_2_ in the FG partition is constrained to be equal to 1. The positions of codons detected as positively selected are indicated with their posterior probability. BG, background partition; BrS, branch-site; d_N_/d_S_, ratio of nonsynonymous to synonymous substitutions; FG, foreground partition; lnL, model likelihood; LRT, the *D* value of a likelihood ratio test; np, number of model parameters; *p*-value, the *p*-value derived from a χ^2^ distribution with 1 degree of freedom; TULV, Tula orthohantavirus; TULV-CEN.S, Central South TULV; TULV-EST.S, Eastern South TULV; κ, transition to transversion ratio.
(XLSX)

S5 Table Results from evolutionary analyses performed in the software HYPHY. TULV genome segments were analyzed separately using the FUBAR and MEME methods to detect amino acid positions under natural selection. Positively selected positions are indicated with the posterior probability *P* (for FUBAR) or a *p*-value (for MEME). TULV, Tula orthohantavirus.
(XLSX)

S6 Table Results of site models implemented in the software PAML fitted to individual TULV genome segments. Nonsynonymous to synonymous substitutions ratios (*ω* = d_N_/d_S_) are shown for each site class for models M0–M3 (*ω*_0_–*ω*_2_). For models M7–M8, the shape parameters *p* and *q* describing the beta distribution are indicated as well as *ω*_p_ for the positively selected site class in models M8 and M8a. The proportions of sites in each site class are listed in parenthesis. Model likelihoods were compared to a null model using an LRT. df, degrees of freedom; lnL, model likelihood; LRT, the *D* value of a likelihood ratio test; np, number of model parameters; *p*-value: the *p*-value derived from a χ^2^ distribution; *κ*, transition to transversion ratio; TULV, Tula orthohantavirus.
(XLSX)

S7 Table Reference sequences for TULV clades in the order of appearance in the phylogenetic trees (Fig 2, S3 and S4 Figs) from top to bottom and PUUV outgroup strains. Columns indicate the genome segments, GenBank accession numbers, and TULV clade associations. PUUV, Puumala orthohantavirus; TULV, Tula orthohantavirus.
(XLSX)

## Abbreviations

CDS: coding sequence
d_N_/d_S_: ratio of nonsynonymous to synonymous substitutions
GPC: glycoprotein precursor
L-segment: large genome segment
MCMC: Markov chain Monte Carlo
MHC: major histocompatibility complex
M-segment: medium genome segment
mtDNA: mitochondrial DNA
nucDNA: nuclear DNA
PUUV: Puumala orthohantavirus
S-segment: small genome segment
TULV: Tula orthohantavirus
TULV-CEN.S: Central South TULV
TULV-EST.S: Eastern South TULV
